# Epidemiology and prevention of respiratory syncytial virus infections in children in Italy

**DOI:** 10.1186/s13052-021-01148-8

**Published:** 2021-10-02

**Authors:** Chiara Azzari, Eugenio Baraldi, Paolo Bonanni, Elena Bozzola, Alessandra Coscia, Marcello Lanari, Paolo Manzoni, Teresa Mazzone, Fabrizio Sandri, Giovanni Checcucci Lisi, Salvatore Parisi, Giorgio Piacentini, Fabio Mosca

**Affiliations:** 1 Department of Health Sciences, Section of Pediatrics, University of Florence, Florence, Italy; 2 Department of Woman’s and Child’s Health, University Hospital of Padova, Padova, Italy; 3 Department of Health Sciences, University of Florence, Florence, Italy; 4 Pediatric Disease Unit, Bambino Gesù Children Hospital IRCCS, Piazza Sant’Onofrio 4, 00165 Rome, Italy; 5 Department of Public Health and Pediatric Sciences, Complex Structure Neonatology Unit, University of Turin, Turin, Italy; 6 Department of Medical and Surgical Sciences, Alma Mater Studioru, University of Bologna, Bologna, Italy; 7 Department of Pediatrics and Neonatology, University Hospital Degli Infermi, Biella, Italy; 8 ASL RM 1, Rome, Italy; 9 Neonatal Intensive Care Unit, Maggiore Hospital, Bologna, Italy; 10 Sanofi Pasteur, Medical Affairs, Milan, Italy; 11 Department of Pediatrics, Pediatric Division, University Hospital of Verona, Verona, Italy; 12 NICU Fondazione IRCCS Ca’ Granda Ospedale Maggiore Policlinico, Milan, Italy; 13 Department of Clinical Sciences and Community Health, University of Milan, Milan, Italy

**Keywords:** Respiratory syncytial virus, RSV, RSV epidemiology, RSV pediatric burden, RSV prevention, LRTI, RSV vaccines, Monoclonal antibodies

## Abstract

Respiratory syncytial virus (RSV) is the leading global cause of respiratory infections in infants and the second most frequent cause of death during the first year of life. This highly contagious seasonal virus is responsible for approximately 3 million hospitalizations and 120,000 deaths annually among children under the age of 5 years. Bronchiolitis is the most common severe manifestation; however, RSV infections are associated with an increased long-term risk for recurring wheezing and the development of asthma. There is an unmet need for new agents and a universal strategy to prevent RSV infections starting at the time of birth. RSV is active between November and April in Italy, and prevention strategies must ensure that all neonates and infants under 1 year of age are protected during the endemic season, regardless of gestational age at birth and timing of birth relative to the epidemic season. Approaches under development include maternal vaccines to protect neonates during their first months, monoclonal antibodies to provide immediate protection lasting up to 5 months, and pediatric vaccines for longer-lasting protection. Meanwhile, improvements are needed in infection surveillance and reporting to improve case identification and better characterize seasonal trends in infections along the Italian peninsula. Rapid diagnostic tests and confirmatory laboratory testing should be used for the differential diagnosis of respiratory pathogens in children. Stakeholders and policymakers must develop access pathways once new agents are available to reduce the burden of infections and hospitalizations.

## Introduction

Respiratory syncytial virus (RSV) is the most common pathogenic agent responsible for respiratory infections in children up to the age of 2 years and causes a wide range of clinical manifestations, including upper respiratory tract infections (URTIs) and lower respiratory tract infections (LRTIs) [1]. More than 60% of all children are infected by RSV within 1 year after birth and nearly all children are infected by RSV at least once within 2 years after birth [2, 3].

In children below the age of 1 year, RSV represents the second cause of death globally after malaria, the first cause of death among respiratory infections, and the first cause of hospitalization [4–6]. In 2015, the estimated global impact of RSV infections in children below the age of 5 years was approximately 33 million LRTI episodes (uncertainty range: 21.6–50.3 million), 3.2 million hospitalizations (uncertainty range: 2.7–3.8 million), and 120,000 deaths (uncertainty range: 94,000–149,000) [7].

Recent studies and reviews confirm that the main risk factors for hospitalization due to RSV infection are age < 1 year and birth just before or during the endemic season. Children born healthy at term with no known risk factors account for up to 85% of the children hospitalized due to RSV [8, 9]. Additional risk factors are prematurity and the presence of comorbidities, such as congenital heart disease, chronic respiratory diseases, and immunodeficiency. Other factors increasing the risk for RSV infection and related complications include living with older siblings, low birth weight, exposure to cigarette smoke, and lack of breastfeeding [10].

**Fig. 1:**
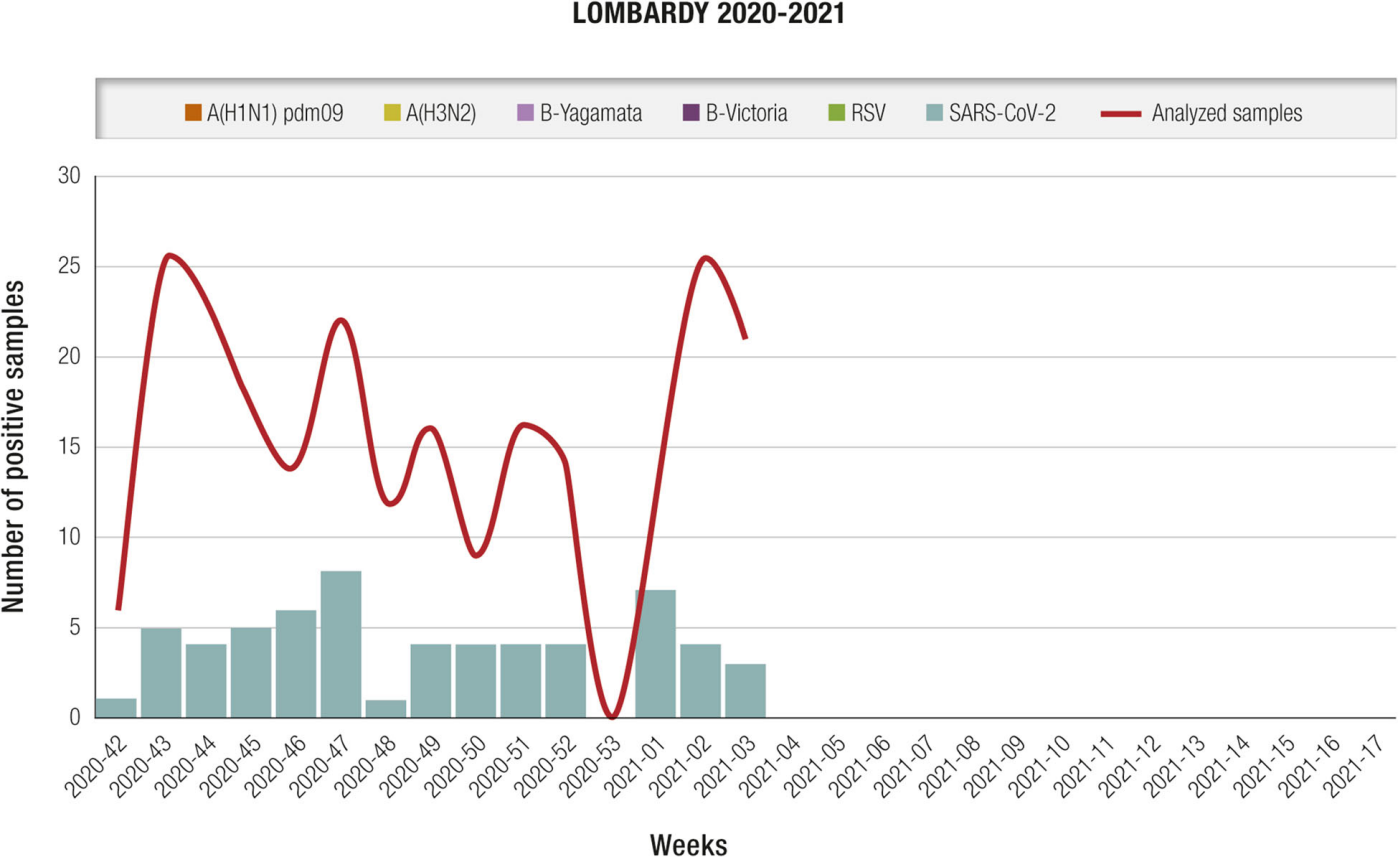
Number of respiratory samples sent by sentinel physicians and number of samples testing positive for influenza virus, SARS-COV-2, and RSV; 2020–2021 season, Lombardy (updated January 27, 2021). SARS-COV-2 was the only virus detected in tested influenza-like illness samples. *RSV* respiratory syncytial virus [12]

RSV is a seasonal virus, characterized by variable epidemiology, depending on geographic area and climate. In temperate regions of the Northern hemisphere, including Italy, virus diffusion generally occurs in the period spanning October/November to March/April, with peak incidence in January/February, which partly overlaps with the influenza virus season [1, 5]. In Italy, restrictive measures for prevention and control of the SARS-COV-2 virus pandemic – especially physical distancing, the use of face masks, and the discontinuation of in-person teaching activities – are likely responsible for the reduced circulation of RSV and other respiratory pathogens during the 2020–2021 season detected by the Italian Influenza Surveillance Network (InfluNet) system [11]. Although specific national data on RSV are not available yet, weekly surveillance reports by the Lombardy region confirmed reduced RSV circulation (Fig. 1) [12] as well as a decreased RSV bronchiolitis hospital admissions in a pediatric tertiary hospital in Rome [13]. SARS-COV-2 was the only virus detected in samples from individuals with influenza-like illness (ILI) tested between October 2020 and January 2021. Although there are strong seasonal trends, RSV circulation may increase in periods outside the typical seasonality; for example, the ILI surveillance system in Australia revealed a higher than expected number of cases during the austral spring, from September to December; in contrast, the winter data indicated that RSV circulation was lower than expected [14–16]. This confirms the importance of year-round surveillance and indicates that removal of pandemic restrictions, especially those on circulation and distancing, promotes the resurgence of RSV, even during inter-seasonal periods.

## Characteristics of RSV

**Fig. 2:**
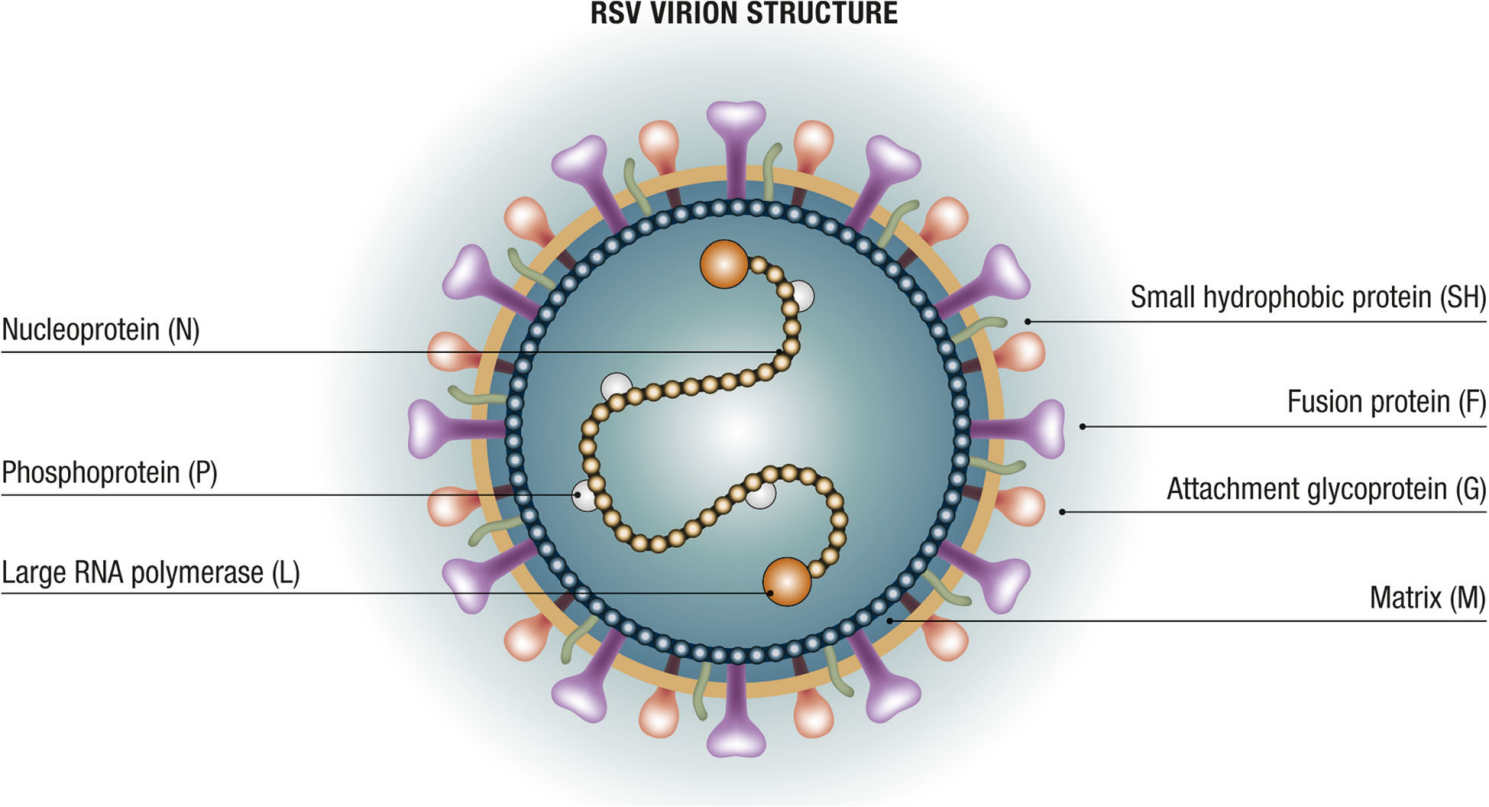
Structure of RSV. *RSV* respiratory syncytial virus. Modified from [16]

RSV is a capsulated pneumovirus of the Paramyxoviridae family with three membrane proteins called small hydrophobic (SH), attachment glycoprotein (G), and fusion protein (F) (Fig. 2). The F and G proteins induce protective neutralizing antibody responses. An effective and timely immune response against protein F can prevent severe manifestations of the infection [17]. Moreover, protein F is characterized by considerable inter-strain conservation, making it is an excellent target for potential vaccines and monoclonal antibodies (mAbs) [18]. Protein G is important for the induction of protective antibodies and the modulation of host immunity. RSV is divided into A and B subtypes based on the protein G sequence. The A subtype appears to correlate with more severe infections. Both subtypes circulate simultaneously during the annual epidemic season, although typically one predominates each year [18, 19].

## Clinical forms of RSV infection: bronchiolitis

RSV is highly contagious, causes frequent re-infections in children – even several times during the same season – and is generally transmitted via direct or indirect contact with oral or nasopharyngeal secretions [5]. The symptomatology of an RSV infection generally starts after a 4–6 day incubation period, with flu-like symptoms and manifestations affecting the upper airways, such as nasal congestion, rhinorrhea, and cough. In neonates and children below the age of 2 years, the clinical picture may evolve into bronchiolitis, the most common clinical syndrome associated with severe RSV infection [1, 10].

Bronchiolitis is an inflammation of the small airways of the lungs occurring after the onset of rhinitis and associated with cough and dyspnea; younger children may also experience fever, feeding difficulties, and irritability [20]. In neonates and especially infants, RSV infection may be associated with other clinical manifestations, such as pneumonia and wheezing (i.e., whistling sound during breathing) [21].

It is now known that RSV morbidity extends beyond the acute episode; RSV infections occurring during the first year of life, including those not requiring hospitalization, are associated with an increased risk for recurring wheezing and the development of asthma [20–22]. A Scottish study in over 740,000 neonates followed to age 18 years, revealed that children hospitalized for RSV infections during the first 2 years of life had a three-fold higher risk of hospitalization for asthma, and used significantly more anti-asthmatic drugs compared with controls [23]. It is not clear whether this association is due to persistent pulmonary damage caused by RSV or whether an underlying lung condition predisposes children to both RSV infection and the onset of other respiratory disorders; however, avoiding chronic respiratory morbidity should be considered among the potential advantages of strategies to prevent RSV infections in the neonatal and pediatric age groups [24].

## Treatment and prevention of RSV infections

To date, no effective and specific treatment is available to cure RSV infections and RSV-specific antiviral agents are still undergoing preliminary research. Mild RSV infections are treated symptomatically, whereas more severe forms such as bronchiolitis require supportive measures, such as oxygen, hydration, and nutrition. International and Italian guidelines provide precise indications for managing bronchiolitis in hospital or across the national territory [25, 26].

Because of the lack of an effective therapy, reduction of morbidity and mortality from RSV must rely on preventive measures. The only agent currently available to prevent RSV infections is palivizumab, a mAb directed against an antigenic site on protein F. The use of palivizumab is regulated by directives from the Italian Medicine Agency (AIFA), which are based on evidence from the literature and recommendations by neonatology and pediatric scientific societies, as well as on the Summary of Product Characteristics of palivizumab [27–29]. Accordingly, palivizumab is reimbursed by the National Health Service, through a therapeutic plan, only for children born preterm at ≤35 weeks gestational age and who are less than 6 months old at the onset of the seasonal RSV epidemic (November), and for children who are < 2 years of age and have major risk factors, such as bronchopulmonary dysplasia, congenital heart disease with pulmonary hypertension, or cyanotic heart disease [28, 30]. Nevertheless, some regions of Italy may apply more selective reimbursement criteria. The use of palivizumab is restricted by not only the narrow therapeutic indication but also the high cost and the need for monthly administrations during the epidemic period because of its short half-life [31].

Research in the field has been driven by the need for safe and effective preventive tools against RSV infection that provide durable and sustainable protection. The development of vaccines or mAbs against RSV has been included among the priorities of the World Health Organization (WHO) [32]. To date, 38 candidates comprising vaccines and mAbs are in clinical trials; several are expected to be available within the next 5 years [18, 33].

In the future, vaccines and mAbs against RSV will be available and accessible globally. Meanwhile, an in-depth assessment of RSV epidemiology is required to determine the seasonality of transmission in different geographic areas, and dissemination in different age and risk groups. Surveillance systems must be implemented or strengthened to support the establishment of prevention policies and related access pathways to immunization against RSV. Having vaccines and mAbs available to prevent infectious diseases is among the recommendations issued by the Italian Ministry of Health National Immunization Technical Advisory Group for the drafting of the next National Vaccine Plan [34, 35].

## Epidemiological surveillance

The establishment of a universal pediatric RSV prevention strategy in Italy requires reliable precise epidemiological data on infection seasonality; these data will be critical for monitoring the impact of the immunization programs introduced. Epidemiological evidence on RSV in Italy is currently inadequate because surveillance is not conducted continuously, systematically, and homogeneously across the national territory. The available data are from local, single-center, ad-hoc studies with limited observation periods.

The WHO has recently implemented a Global Respiratory Syncytial Virus Surveillance effort, integrating it into the existing Global Influenza Surveillance and Response System. Results from the first phase confirmed the feasibility of basing RSV surveillance on existing influenza surveillance systems, with a marginal cost increase and no significant negative impact on influenza surveillance [36, 37]. The WHO recommends acute respiratory infection (ARI) as the most appropriate definition for identifying cases of RSV infection in the context of this system and in community surveillance [38]. This differs from the ILI definition recommended for identifying suspected cases of influenza. To identify hospitalizations suspected to be caused by RSV, the WHO proposes using the severe ARI (SARI) and LRTI definitions, which also include bronchiolitis and pneumonia [38].

Starting with the 2019–2020 season, Italian national community-based RSV surveillance has been conducted through InfluNet, which has been active for several years and uses the ILI case definition [39]. InfluNet is based on a network of sentinel general practitioners and primary care pediatricians who report suspected ILI cases in their practice and send biological samples for pathogen identification. Virologic analyses are performed by InfluNet laboratories and the National Institute of Health itself, which coordinates the surveillance system on behalf of the Ministry of Health. Thus, RSV surveillance has been initiated, but is based on the ILI case definition rather than the more appropriate ARI definition. Starting in the 2020–2021 season, the InfluNet system has been further integrated to include the CovidNet system for the surveillance of the SARS-COV-2 pandemic virus. Its yearly activities start in the 42nd week (mid-October) and last until the 17th week (end of April) of the following year [40]. However, reports from InfluNet on the first season (2019–2020) and the current season (2020–2021) do not provide data on RSV circulation [39, 40].

Table 1 reports the case definitions recommended by the WHO and used in Italy for community- and hospital-based RSV surveillance [38, 39].

**Table 1:** Case definitions for community- and hospital-based RSV surveillance: Italy and WHO recommendations [36, 37]

Case definition	Criteria
ILI	Measured body temperature ≥ 38C°
	Cough
	Acute – defined as onset within the past 10 days
ARI	Acute – defined as sudden onset of symptoms
	Respiratory infection – defined as having at least one of the following:
	Shortness of breath
	Cough
	Sore throat
	Coryza
SARI	Severe – defined as requiring hospitalization
	Acute – defined as onset within the past 10 days
	Respiratory infection, with cough and/or shortness of breath
	In infants < 6 months old, possible apnea and/or sepsis
LRTI	LRTI, which can affect the bronchi, the bronchioles, and the lungs: bronchitis, bronchiolitis, pneumonia
	No standard definition for its identification

*ARI* acute respiratory infection, *ILI* influenza-like illness, *LRTI* lower respiratory tract infection, *RSV* respiratory syncytial virus, *SARI* severe acute respiratory infection, *WHO* World Health Organization

**Fig. 3:**
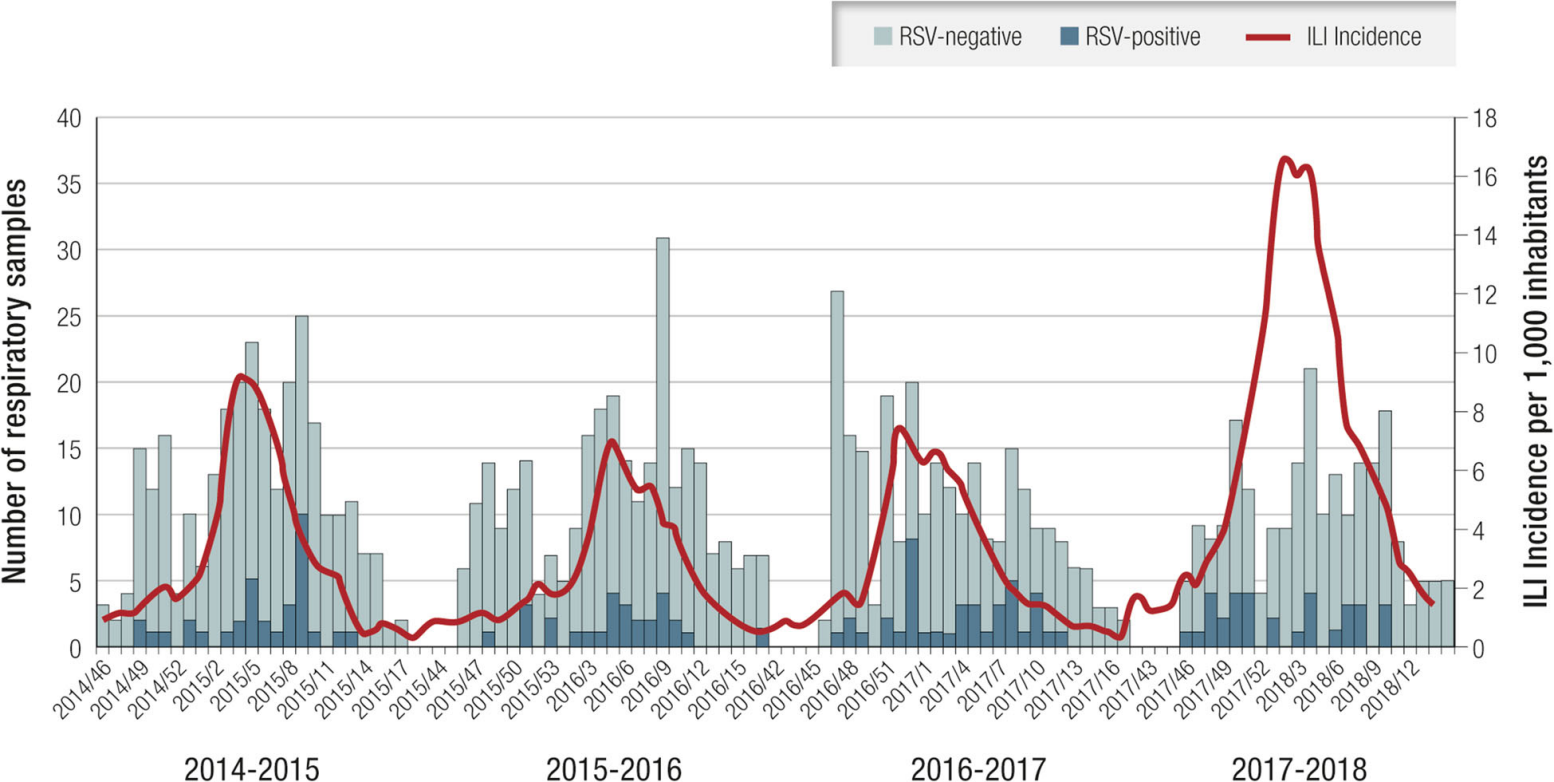
Number of RSV-positive and RSV-negative samples and ILI incidence per 1000 residents per week in four consecutive seasons. *ILI* influenza-like illness; *RSV* respiratory syncytial virus. Modified from [37, 39]

The seasonality of RSV infections in Lombardy over four consecutive winter seasons (2014–2018) was identified through community-based surveillance (Fig. 3). During the study period, RSV circulated from mid-November until the end of April, peaking in mid-February (median: week 8; range: 6–10); the duration of RSV circulation was about 4 months (median: 15.5 weeks; range: 13–19 weeks). This is consistent with the results of a previous analysis of data from 15 European countries, which did not include Italy [41].

RSV was detected in all age groups; however, the RSV positivity rate among ILI cases was highest in the pediatric age group ≤5 years (27.8%; 51/183) (Table 2) [41].

**Table 2:** Number of ILI cases and ILI RSV-positive cases, per age group during four consecutive seasons in Lombardy [39]

Age groups	ILI cases, n (%)	RSV-positive ILI cases, n (%)
0–5	183 (17.5)	51 (27.8)
6–15	158 (15.1)	30 (18.9)
16–45	332 (31.7)	16 (4.8)
46–65	249 (23.8)	23 (9.3)
> 65	125 (11.9)	15 (12)
Total	1047 (100)	135 (12.9)

*ILI* influenza-like illness, *RSV* respiratory syncytial virus

To date, there is no Italian national system for surveillance of hospitalizations suspected to be due to RSV, which should be implemented using SARI and LRTI case definitions; however, hospitalization surveillance studies have monitored and analyzed SARIs and LRTIs in defined geographic areas and time periods [1, 42–44].

**Fig. 4:**
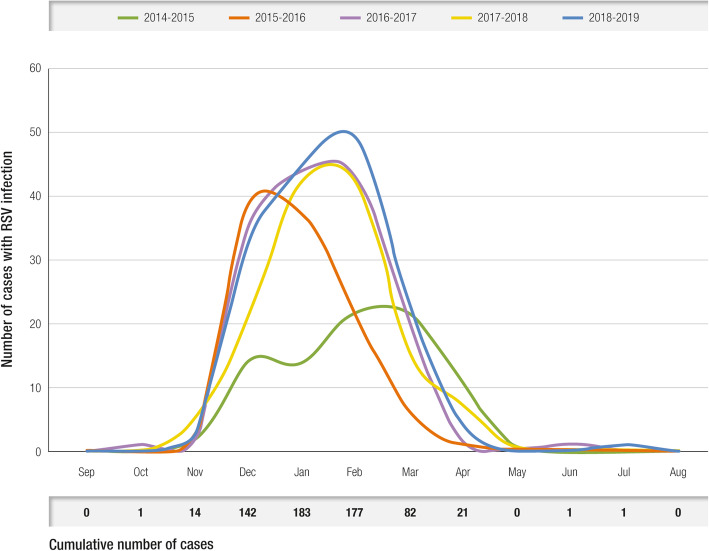
Seasonal trend of pediatric hospitalizations due to respiratory infections from RSV in Tuscany, 2015–2019. *RSV* respiratory syncytial virus [1]

A large retrospective study in Tuscany, identified 1627 children aged 0–6 years who were hospitalized between 2014 and 2019 due to a respiratory infection; of those, 624 (38.4%) were RSV-positive based on polymerase chain reaction assays on nasopharyngeal swab samples. Most of the children had been born at term (471/624; 75%) or late preterm between 34 and 37 weeks of gestation (111/624; 18%), confirming that severe RSV infections are not limited to infants with moderate to severe prematurity, who accounted for only 7% of patients hospitalized due to RSV (42/624) in this study. The study confirms that the time distribution of hospitalizations due to RSV coincides with the seasonality observed in community surveillance studies conducted in other Italian regions (i.e., November to April); 94% of RSV-positive children (584/624) were hospitalized between December and March (Fig. 4) [1].

The main risk factors for RSV infection in the pediatric age, based on the international literature and confirmed by the above-mentioned Italian studies, are seasonality (in Italy corresponding to November to April) and age, especially < 1 year, with higher risk during the first 3–6 months of life. Concerning gestational age at birth, although severe prematurity is an important risk factor, children born late preterm and at term are at risk of infection and, in absolute terms, account for the largest proportion of hospitalizations due to RSV.

It is important to determine the RSV infection incidence in children in the community and prevent nosocomial diffusion by performing rapid diagnostic tests and confirmatory laboratory tests for the differential diagnoses of ARIs and SARIs, respectively [45]. In the current scenario, where SARS-COV-2 and RSV co-circulate, differential diagnosis through rapid testing and confirmatory laboratory tests represents an additional tool to monitor the circulation of respiratory pathogens more accurately in children.

There are currently no Italian networks or national registries providing a precise estimate of the economic and social impact of RSV infection in neonates and children. Their establishment in the near-term would support cost-effectiveness analyses to inform decisions on the implementation of new vaccines or mAbs. Recently, a study conducted at the Bambino Gesù Children’s Hospital in Rome evaluated hospitalization costs associated with bronchiolitis in children aged 1 month to 1 year admitted in 2017. Of 531 children admitted with bronchiolitis, 58% (310/531) were RSV-positive, confirming the previous finding that RSV is the leading etiologic cause of bronchiolitis in this age group. The total hospitalization cost was nearly 3 million Euros; of which nearly 1.8 million Euros was accounted for by hospitalizations due to RSV. The average cost per hospitalized patient was significantly higher (*p* = 0.04) in RSV-positive cases (5753 ± 2042 Euros) compared with cases due to other pathogens (5395 ± 2041 Euros), a higher median cost due also to the significatively longer hospital stay (*p* < 0.001) for RSV-positive cases (4.98 ± 2.18 days) compared with those infected by other pathogens (4.22 ± 2.16 days; Table 3) [46].

**Table 3:** Hospitalizations due to bronchiolitis from RSV versus bronchiolitis from other pathogens, Bambino Gesù Children’s Hospital, 2017. Data shown as mean (standard deviation) [44]

	RSV (*n* = 310)	Other pathogens (*n* = 217)	*p* value
Age, days	77.98 (± 58.02)	78.80 (± 64.16)	NS
LOS, days	4.98 (± 2.18)	4.22 (± 2.16)	< 0.001
Total cost, million Euro	1.78	1.17	
Mean cost, Euro	5753 (± 2041)	5395 (± 2040)	0.04

*LOS* length of stay, *NS* not significant, *RSV* respiratory syncytial virus

## Future scenarios for prevention

The above evidence demonstrates the importance of protecting all neonates and children up to 1 year of age throughout the RSV epidemic season, which usually lasts 5–6 months from November to April in Italy. However, the only agent currently approved for the prevention of RSV, palivizumab, is indicated only for preterm infants born at ≤35 weeks of gestation. No agents are approved for healthy infants born at term or late preterm, which together account for up to 95% of all newborns.

When new mAbs and vaccines to prevent RSV infections are available, prevention policies must consider the seasonal characteristics of the virus, to ensure that all neonates and children below the age of 1 year, regardless of gestational age at birth, are protected throughout the seasonal period when RSV circulation is highest.

To contribute to a significant reduction in the burden of infections and hospitalizations, future vaccines and mAbs against RSV must provide protection that is more extended over time and available for use in all neonates and infants [19, 33, 47].

The new approaches currently being tested include maternal vaccines for pregnant women to protect neonates during the first months after birth through transplacental transfer of maternal antibodies; mAbs to provide immediate protection for up to 5 months after administration, for use in all neonates and infants during the first months of life; and pediatric vaccines that could be administered to infants a few months after birth to provide immunity lasting for years [19, 33, 47].

The main approaches currently being investigated and developed are summarized in Table 4 [5, 6, 17–19, 32, 33, 47].

**Table 4:** Characteristics of vaccines and mAbs against RSV in the Italian epidemiological context [5, 6, 15–17, 30, 31, 45]

Type	Target population and main characteristics
Maternal vaccines	Vaccination of pregnant women to protect neonates through transplacental transfer of maternal antibodies
	Protection of the neonate from birth
	Duration equivalent to the life of maternal antibodies (i.e., 2–4 months)
	Can immunize only infants born just before and during RSV epidemic season, and that have been born at term
mAbs with extended half-life	Neonates and infants
	Immediate onset of protection
	Duration of up to 5 months, throughout the RSV season
	Can immunize all children, at birth if born during the RSV season and by appointment if born before the season
Pediatric vaccines	Children, early infancy
	No protection during the first months of life
	Durable protection (years) throughout childhood

*mAb* monoclonal antibody, *RSV* respiratory syncytial virus

These strategies should be evaluated in the context of universal coverage for this age group, including children born at term and healthy preterm births, thereby overcoming the current limitations of palivizumab use. Besides reducing the risk of acute RSV infections, a universal approach to prevention would also reduce the risk of medium- and long-term consequences, such as recurrent wheezing and asthma during childhood, which may require further hospitalizations and medication use [24]. Prevention of neonatal RSV infections with palivizumab was associated with a reduction in the occurrence of such events in the medium to long term. Therefore, evaluation of the potential impact of new vaccines and mAbs should consider the possibility of reducing recurring wheezing and asthma episodes and the associated hospitalizations and medication use, which represents an important novel aspect [17, 24, 48]*.*

Another challenge to the effective implementation of RSV prevention is the establishment of appropriate clinical pathways for each agent. This will require identification of healthcare facilities and clinical professionals for drafting recommendations, prescribing, and administering the new agents. From a practical perspective, strategies must be differentiated according to birth date into neonates born during or just before the RSV epidemic season, and neonates born outside the RSV epidemic season.

### Maternal vaccination

**Fig. 5:**
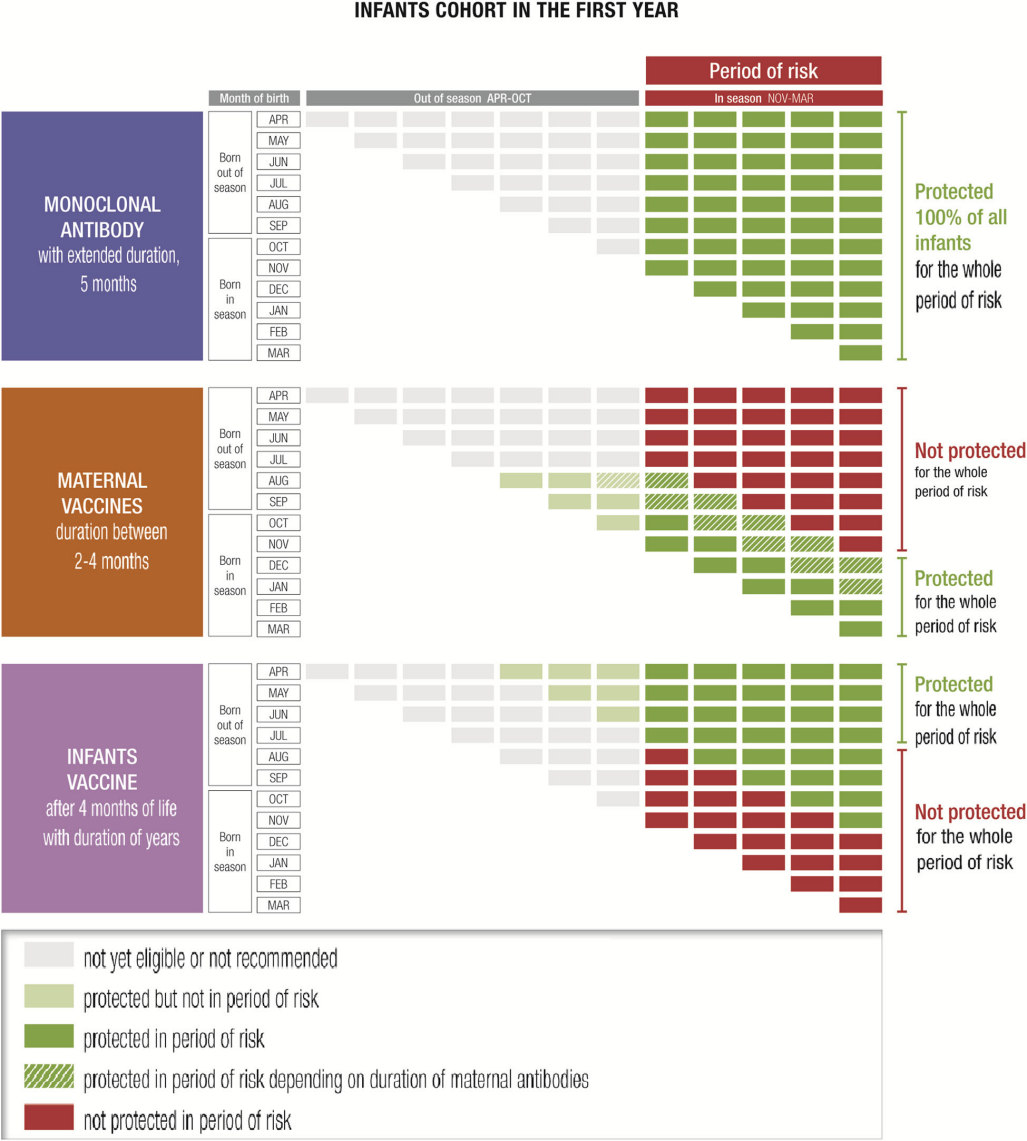
Periods of potential protection from RSV infection using different prevention strategies, based on RSV seasonality in Italy. *RSV* respiratory syncytial virus. Modified from [5]

A maternal RSV vaccination strategy might follow the pathway for vaccinating pregnant women against influenza and pertussis already recommended in the National Vaccine Plan 2017–2019, administering the RSV vaccination during the sessions scheduled for those vaccinations [49]. The maternal vaccination strategy would be a valid option only for children whose birth is expected to occur just before the RSV epidemic season, but not for those born in the previous months. Moreover, the real efficacy of the vaccine will depend on whether it is administered in compliance with the approved indication in terms of gestational period, and whether gestation ends at term. The duration of protection would be determined by the above-described conditions and the duration of maternal antibodies, and thus will likely range from 2 to 4 months (Fig. 5).

### Monoclonal antibodies

A prevention strategy based on passive immunoprophylaxis with single doses of an extended half-life mAb could last up to 5 months and cover the entire RSV season. Administration could take place during the pre-discharge visit of the neonate. Other pre-discharge preventive interventions, such as the administration of vitamin K and antimicrobial eyedrops, are routinely planned [50]. The anti-RSV mAb could be administered in this setting to all neonates born after the onset of the epidemic season. Children born before the RSV season who are < 1 year old at its onset would be considered at-risk and could be contacted and scheduled to receive immunoprophylaxis. In this case, the feasibility and appropriateness of administering the mAb at a health supervision visit or at a visit scheduled on the pediatric vaccine calendar should be assessed; both are routine visits, which could be scheduled to take place before the onset of the RSV season [49, 51]. Another option could be to administer the mAb at a hospital-based outpatient clinic, as is already done for palivizumab-eligible premature infants, who are regularly contacted to schedule their monthly dose (Fig. 5).

### Pediatric vaccines

Pediatric vaccines could be indicated in all children during infancy but would not cover the first months of life, as they cannot be administered at birth. When the gap in protection coincides temporally with RSV seasonality, the infant would be exposed to the risk of infection and complications. Thus, this strategy should be implemented together with one of the two approaches discussed above (i.e., preceded by either maternal vaccination or administration of a mAb). Administration could be included in the existing vaccine schedule [49]. The duration of protection could extend throughout childhood and therefore have strategic importance for limiting RSV circulation among children (Fig. 5).

## Conclusions

The evaluation of new vaccines and mAbs should consider not only the current unmet medical need to prevent RSV disease in all neonates and children but also the direct and indirect healthcare costs related to RSV infection, such as the costs for access to pediatric primary care, emergency departments, and possible hospital admissions associated with an acute event, as well as any additional direct or indirect costs for hospitalizations and medications due to recurring wheezing and asthma that may occur in the medium to long term.

Epidemiological data on RSV In Italy, although limited to a few geographic areas and observation periods, is in line with international evidence showing that pediatric RSV infection has a significant impact on morbidity.

To better define the burden of RSV in Italy, it would be appropriate to implement and strengthen:

- Community-based epidemiological surveillance of RSV infections in the pediatric population, especially up to the age of 2 years, leveraging the currently implemented InfluNet influenza-monitoring protocol, and providing reports describing RSV-positive cases identified in this age group using the ARI case definition rather than the ILI case definition
- Surveillance of hospitalizations due to respiratory infections, using the SARI and LRTI case definitions, in children up to the age of 2 years
- Medium- to long-term follow-up to assess the onset of wheezing and asthma in subjects who experienced an RSV infection while aged 0–2 years. These data will provide a baseline for comparison when RSV vaccines and anti-RSV mAbs are available

In parallel, stakeholders and policymakers should establish pathways for access to RSV prevention. New and innovative options for prevention, such as long-acting vaccines and mAbs, should be used:

- To prevent RSV infections in all neonates and children < 1 year of age, regardless of their gestational age at birth
- To protect all neonates and children throughout the RSV epidemic season (November to April in Italy)

Prevention strategies should consider the type and characteristics of available agents:

- Vaccines for pregnant women and mAbs, which can be used to protect neonates from birth and during the RSV epidemic season
- Pediatric vaccines, which can be administered to all children during infancy to achieve longer-term protection and thus limit RSV circulation in the pediatric population
- The duration of the protection provided and the period when the birth is expected to occur should both be considered for identifying the most appropriate products to implement a universal strategy for neonates and children during their first RSV season
- The possible synergy between maternal vaccination or prophylaxis with a mAb, to protect neonates from birth, and subsequent pediatric vaccination to achieve more durable protection and limit RSV circulation

The availability of novel, effective tools to prevent RSV infections makes the collection of epidemiological data in Italy crucial. These data should inform decisions on appropriate prevention strategies and the establishment of clinical pathways for each new agent, considering RSV seasonality and healthcare system organization.

Finally, the economic impact of RSV infection in neonates and children must be evaluated in terms of both direct and indirect burden on health resources and services, including visits to primary care pediatricians, accesses to emergency departments, and hospital admissions due to acute RSV infections, as well as resource use in subsequent years, such as additional hospital admissions and medication use due to wheezing or the onset of asthma. These data, together with the cost and effectiveness of each new agent, can inform cost-effectiveness analyses and a broader assessment of the value of RSV prevention.

## Data Availability

Not applicable.

## Abbreviations

AIFA: Italian Medicines Agency
ARI: Acute respiratory infection
F: Fusion protein
G: Attachment glycoprotein
ILI: Influenza-like illness
LRTI: Lower respiratory tract infection
mAb: Monoclonal antibody
RSV: Respiratory syncytial virus
SH: Small hydrophobic (protein)
URTI: Upper respiratory tract infection
WHO: World Health Organization
